# Genetic Diversity within Snap Beans and Their Relation to Dry Beans

**DOI:** 10.3390/genes9120587

**Published:** 2018-11-28

**Authors:** Lyle Wallace, Haidar Arkwazee, Kelly Vining, James R. Myers

**Affiliations:** 1 Department of Horticulture, Oregon State University, 4017 Ag & Life Sciences Bldg., Corvallis, OR 97331, USA; Kelly.Vining@oregonstate.edu; 2 Department of Horticulture, University of Sulaimani, P.O. Box 334 Sulaimani, Iraq; arkwazee1983@gmail.com

**Keywords:** *Phaseolus vulgaris*, common bean, green bean, phylogenetics, single nucleotide polymorphism, principal components, Structure, K-means clustering, expected heterozygosity, gene pools, domestication

## Abstract

Two hundred forty-six snap bean genotypes and 49 dry beans representing both centers of domestication and six bean races with materials from Europe, Asia, and the Americas were genotyped using a single nucleotide polymorphism (SNP) array. The data was analyzed for expected heterozygosity, K-means clustering, principal components, phylogenetic relationships, and population substructure. When all gene pools of snap bean were assembled, the expected heterozygosity was roughly equivalent to a carefully chosen panel of dry beans representing all bean races and centers of domestication demonstrating the genetic richness of snap materials in total. K-means clustering and *K* = 2 structure analysis showed significant mixing of gene pools in the European and American commercial snap materials and the dominance of the Andean center of domestication among commercial contemporary snap beans. Conversely, the same analysis showed that Chinese, Iberian, and heirloom materials were underrepresented in contemporary materials. Further, Structure analysis revealed eight distinct groups within snap beans. Two showed strong kinship to the Middle American center of domestication, three to the Andean center of domestication, and three showed admixture between the two centers. Snap beans may have been independently derived from dry beans more than once and from both centers. Overall, we identified eight potential germplasm pools for snap bean.

## 1. Introduction

*Phaseolus vulgaris* appears to have split from its nearest relatives approximately 1.3 million years ago (MYA) based on ribosomal internal transcribed spacer sequence data [1]. The available evidence strongly points towards Middle America as the ultimate origin of *P. vulgaris* where all the nearest relatives of *P. vulgaris* are also localized. This origin hypothesis has been complicated by new evidence that *P. vulgaris* may have originated in Middle America but the ancestral form of the species underwent further speciation through allopatry in the Amotape–Huancabamba Depression of the Andes [2]. Several complicated movements of germplasm followed this speciation event culminating in the development of two separate gene pools in the Andes and Middle America at ~165,000 BP [3]. Humans then domesticated *P. vulgaris* within each of these gene pools nearly simultaneously in a dual event at ~8000 BP. These concurrent domestications of the same species in two geographically distinct regions has resulted in a great phylogenetic divide in domesticated beans whose existence is supported by evidence from multiple sources. The consequences of this separation are numerous, including distinct adaptations and traits in each gene pool, and genetic incompatibilities between the gene pools. Seed size, heliotropism, leaf size, and phaseolin storage protein type are some of the characteristics strongly influenced by ancestry. Plant breeders have also observed that characteristics critical to breeding, such as higher yields and stress tolerances, are highly modulated by center of domestication [4].

One consequence of this divide is the production of so called bean races distinctive to each gene pool associated with its respective center of domestication that were first described by Singh and colleagues in 1991 [5]. This work to define finer scale gene pools within the larger Andean-Middle American framework began with observations of phenotypic characteristics but was later supported by molecular and biochemical evidence. Specifically, differences in the leaf morphology, seed size, seed shape, pod morphology, stem thickness, internode length, plant habit, allozyme type, and phaseolin type could be used to identify three Mesoamerican races (Durango, Jalisco, and Mesoamerica) and three Andean races (Peru, Chile, and Nueva Granada). The six races could not be identified with absolute reliability and exceptions to both molecular characterization and phenotypic traits did occur in the data, but at a relatively low rate. A seventh race (Guatemala) within the Middle American gene pool, was later distinguished using random amplified polymorphic DNA (RAPD) data and geographic and morphological differences [6]. An additional four studies have shown distinctive clustering of the races using microsatellite data, although only the RAPD work of Beebe has clearly separated race Jalisco from race Durango [7,8,9,10]. All of this molecular research has shown variable overlap between the races, thus underlining the need for further research into bean races.

The single most important event after the speciation and domestication of *P. vulgaris* has been the Columbian Exchange and the dispersal of domesticated beans around the globe forming secondary centers of diversity. The Columbian Exchange brought *P. vulgaris* and other crops to the Iberian Peninsula, Italy, and to the far-flung colonies of Spain and Portugal, such as the Philippines, and sub-Saharan and North Africa. An early Spanish colony that received goods from the Americas was the Philippines, and an obvious port of call near the Philippines would have been the Portuguese colony of Macau, which is an autonomous state in modern day China. As for the remainder of Europe, it is evident that multiple introductions took place over time and a simple lineage through the Iberian Peninsula cannot be derived [11]. One result of this early dispersal of *P. vulgaris* worldwide, however it may have happened, is the development of potential secondary centers of diversity in China and Europe [11,12]

The place of snap beans in this history of domestication and dispersal is unclear as to its initial origins. Superficially, snap beans are dry beans that have fleshier pods that are low in fiber where immature pods are eaten as a vegetable. However, there is a wide range in variation in what are classified as “snap beans”. This variation is reflected in the names that are used for this class around the world. Terminology includes “French bean”, often used in Europe to denote this type, “string bean”, referring to types where the pod suture strings must be removed, and “green bean”—vegetable forms with succulent green pods as opposed to “wax bean” with yellow pods [13]. Those showing the least amount of change from dry beans are the large flat podded types represented by Romano beans in European culture. These have thin pod walls with low fiber at maturity (Table 1).

In addition to low pod wall fiber, many forms of string and snap beans have thick and fleshy pod walls. Contemporary cultivars have pods that are elongated and cylindrical in shape, which also affects seed shape. Another trait now nearly ubiquitous in snap beans is a lack of a “string” (fiber) in the abaxial and adaxial pod sutures. Because both stringy and stringless types occur, genetic control of strings is thought to be independent of pod wall fiber. In this paper, we use the term “snap bean” to generally designate common bean types with low fiber that are used as a vegetable. Contemporary snap beans have been selected for additional traits that adapt them to particular production systems and uses. These include determinant bush (type I) growth habit, prolific flower production combined with concentration of pod set, pods of different sizes with pod cross-sections ranging from oval to round, variation in pod color from yellow (wax beans) to very dark green and white flower and seed color. One hypothesis is that snap beans arose from dry beans through a process of stepwise mutation—the first being reduction in pod fiber followed by changes in pod shape and eventually development of stringless types.

The belief that snap beans have been selected out of dry beans after the Columbian Exchange is based in part on the fact that accessions with snap characteristics are very rare in North, Central and South American landrace collections [4,14]. This rarity may be due in part to the low nutritional density of snap beans in comparison to the protein and carbohydrate content of dry beans, along with the storability of dry beans that led most pre-Columbian peoples of the Americas to prefer dry beans. In addition, traits that reduce pod fiber may reduce fitness of snap relative to dry bean. Because loss of pod fiber prevents normal pod dehiscence, snap beans are more difficult to thresh and clean for mature seed. Reduction in pod fiber may also affect ability to fend off plant pathogens.

In pre-Columbian times, there was very likely consumption of immature dry bean pods [5] when pod fiber is low as is found in subsistence cultures around the world today. This consumption typically happens during the “hunger season” when the crop has been planted and is growing, but stored food reserves are depleted. Because the primary use remained the mature and dry seed, little selection for vegetable characteristics has been practiced. Linguistic evidence suggests the vegetable use of common bean, such as the word, exotl, from Nahuatl language of the Aztecs referring to green pods and the word, chaucha, from the Quechua language also referring to green pods [15,16]. This linguistic evidence, may reflect consumption of immature dry bean pods, or, as other authors have observed, it “(…) may show a long-term knowledge of snap beans that was not introduced or re-introduced as modified dry bean germplasm from outside the region (…)” [16]. In addition, one potential Native North American snap bean, ‘Cherokee Trail of Tears’, has been identified [17]. Based on the low-level presence of snap traits in American landrace material and the linguistic knowledge of snap beans by some pre-Columbian people of the Americas, it is possible that snap traits existed in a few of the materials coming from the Americas, but these traits were highly amplified by selection in Europe where snap beans were developed. Snap beans were returned to the Americas with migration of immigrants to the New World. European farmer selected snap materials form the basis of commercial snap beans today, although snap beans may have also been developed separately in China.

Another aspect of the origin of snap beans that remains unclear is the relationship of snap beans to the Middle American and Andean gene pools and races. The original work on bean races categorized all snap beans (gene pools 11 and 12) as races Nueva Granada and Chile of Andean origin [4,5]. Gepts and Bliss [18] found that the majority of beans with low fiber pods from Europe had T phaseolin (associated with the Andean gene pool) but some landraces had S phaseolin (associated with the Middle American gene pool). In the Americas, all snap beans had either T or C phaseolin [19]. Later work using microsatellite data and RAPDs showed apparent hybridization between Middle American and Andean gene pools in snap beans [15,20,21]. More recently, a phylogenetic analysis of both dry and snap beans using a single nucleotide polymorphism (SNP) array placed several snap bean cultivars in an intermediate region, although the snap beans were more heavily skewed towards the Andean gene pool [22]. These data suggest that snap beans may have originated in more than one gene pool, and have undergone extensive intermating.

Historically, an important region for snap bean production in the 20th and 21st centuries has been the Willamette Valley of Oregon, where a distinctive lineage of snap bean is grown that is generally referred to as “Blue Lake”. This snap bean lineage was brought to Oregon from the Blue Lakes region of California early in the 20th century, but its ultimate origin is unclear. It is known for excellent canning and processing characteristics and quality traits that are desirable to consumers as well as the ability to achieve high yields in the field. The origin of this distinctive lineage has been purported to be ‘Scotia’, ‘Genuine Cornfield’, or ‘White Creaseback’ [23].

The objective of the present study of the genetic history and diversity of snap beans was to elucidate the origins, intermixing, gene pools, and, where possible, the bean races associated with snap beans.

## 2. Materials and Methods

### 2.1. Bean Accessions

The plant materials utilized in this study were compiled from multiple sources. The first source of materials was the Common Bean Coordinated Agricultural Project (BeanCAP) snap bean diversity panel [24], consisting of 141 bush habit snap beans and 8 pole habit snap beans that were mostly derived from commercial bean lines in North America and Europe. The second source of materials was an assemblage of 59 Chinese snap bean genotypes obtained from a trip to China in 1991 by Michael Dickson (Cornell University), placed in the United States Dept. of Agriculture National Plant Germplasm System (USDA-NPGS) Plant Introduction collection at Pullman, WA, USA, but left uncatalogued and provided to the Oregon State University (OSU) Vegetable Breeding Program for evaluation. Four of these genotypes were subsequently removed from the study because there was greater than 20% missing data. The third source consisted of 19 Spanish genotypes from the Misión Biológica de Galicia—Consejo Superior de Investigación Científica (CSIC) (Pontevedra, Spain) collection. These 19 genotypes were a subset that possessed edible pod traits from a larger collection of common bean (de Ron, personal communication). Finally, 24 heirloom beans were added from specialized seed catalogues and the family of Harvey Bruxvoort. We included 48 dry bean accessions from the USDA-NPGS Plant Introduction (PI) collection that were accessed through the Germplasm Resources Information Network (GRIN). Except for ‘Pole Blue Lake FM-1’ and ‘Scotia’, all the PI collection lines were chosen based on their bean race typing. The bean races of the PI lines were determined from previous reports in the literature [5,7,9,10,25]. In total, 295 accessions were included in the study with 49 dry beans and 246 snap beans. More information on the genotypes is available in Table S1.

### 2.2. Genomic DNA Extraction and Genotyping

Genomic DNA was extracted in three groups using an adapted Cetyl trimethylammonium bromide (CTAB) protocol [26]. Roughly 0.5 g of tissue from young trifoliate leaves was pulverized in 500 µL of CTAB buffer and then maintained at 65 °C for one hour. The pulverized leaves and CTAB buffer were extracted with 500 µL of chloroform. The supernatant was moved to a separate tube and then precipitated with 400 µL of 76% ethanol and 10% ammonium acetate. After spinning at high speed in a centrifuge, the pellets were air dried and resuspended in 200 µL of TE buffer. The resuspended DNA was then mixed with 8 µg of RNase A and incubated at 37° for one hour followed by extraction with 300 µL of chloroform. The supernatant was moved to a separate tube and precipitated with 15 µL of 3M sodium acetate (pH 5.2) and 300 µL of 95% ethanol. A wash step with 400 µL of 70% ethanol was done. The resulting pellet was air dried and resuspended in 50 µL of TE buffer. An agarose gel quality check was done on 1 µg of each DNA sample. An ND-1000 UV-Vis Spectrophotometer (Thermo Fisher Scientific, Waltham, MA USA) was used to determine DNA concentrations. Race specific PI collection materials, heirloom materials, and eight Spanish lines were extracted in 2018. ‘Acclaim’, ‘BBL274’, ‘Benchmark’, ‘Booster’, ‘Calgreen’, ‘Castano’, ‘Coloma’, ‘Cyclone’, ‘Flavor Sweet’, ‘Fortex’, ‘Kentucky Wonder’, and ‘Mercury’ were extracted in 2013. The remainder of the Spanish lines and the Chinese materials were also extracted in 2013. Finally, all other BeanCAP lines were extracted in 2009.

DNA samples were analyzed using the Illumina Infinium Genechip BARCBEAN6K_3 platform at the USDA Soybean Genomics and Improvement Laboratory (Beltsville, MD, USA). This SNP array consists of 5398 allele-specific probes. The raw data was processed using GenomeStudio (v2.0.4) software (Illumina, San Diego, CA USA). Two marker positions contained greater than 20% missing data and were removed from the study. Heterozygous positions were treated as missing data. Missing data at the genotype level ranged from 0.22% to 28.54%. Four Chinese genotypes with greater than 20% missing data were removed from the study. After these four were removed, the values ranged from 0.22% to 15.45% with an arithmetic mean missing value of 2.19%. No imputation was done.

### 2.3. Statistical Analysis

Expected heterozygosity (HET) values were calculated using the Heterozygosity calculator (HET) function in the R package ‘GeneticSubsetter’ [27]. The data set contained 93 monomorphic markers out of a total of 5396 markers. These monomorphic markers were not removed for the HET function analysis because their effect was negligible.

Basal branching was poorly supported in a phylogenetic tree of the entire population, therefore, limited phylograms were created using Past 3.2 software [28]. Phylograms were generated for select groups of genotypes for which it was informative in determining lineages. In particular, phylogenetic trees of Blue Lake lines, Chinese lines, and Spanish lines clarified the connections between germplasm collections and to race Chile. Only race Chile was included because it was the only bean race with clear phylogenetic connections to snap beans. All other bean races produced more distant connections of an ambiguous nature. A neighbor-joining tree was generated with a Euclidian similarity index. A 1000 bootstrap procedure was then run. Bootstrap resamples recovered at each node were reported as a percentage. The tree examining Chinese and Spanish materials also contained ‘Aunt Ada’ and ‘Cherokee Trail of Tears’ because these two American heirloom cultivars have shown very little intermixing in a *K* = 2 Structure analysis, are closely associated with either race Chile (‘Aunt Ada’) or race Mesoamerica (‘Cherokee Trail of Tears’), are closely associated with dry beans, and yet they are true snap beans.

A principal coordinates analysis (PCoA) was conducted in Past 3.2 software generating eigenvalues for 295 axes. A Euclidian similarity index was utilized with a *c* = 2 transformation exponent. Nearly half the variation was explained by axes one and two, so a biplot of the first and second axes of the PCoA was generated using XLStat version 2018.5.52447 (Addinsoft, New York, NY USA).

To more closely examine population substructure, both fastStructure and Structure were run. The fastStructure algorithm [29] was run using model components from *K* = 2 to *K* = 9, with a random seed setting of 100. Admixture proportions were visualized using Distruct. Initial *K* values in Structure 2.3.4 software were determined with an admixture model and correlated allele frequencies using a 10,000 burn-in with 50,000 Monte Carlo Markov Chain (MCMC) iterations [30]. The best *K* was identified in fastStructure (*K* = 8) and tested in Structure by calculating L(*K*) from the mean of 20 reps for each value of *K* from 2 to 10. The Wilcoxon nonparametric test of paired levels of *K* from 2 to 10 was used to determine the inflection point for which the likelihood value did not significantly differ between paired levels of *K*. The results from the Wilcoxon nonparametric test was confirmed using the delta *K* method [31]. Both tests suggested that a *K* value of 8 or 9 was optimal. An additional set of runs for *K* = 8 or 9 using a burn-in of 500,000 and 750,000 replicates was conducted. The replicate with smallest likelihood value of 20 replicates (independent runs) was used for analysis. *K* = 2 was also examined because it divided the lines into the two major gene pools of common bean. Bar plots were generated within Structure for *K* = 8.

## 3. Results

### 3.1. K = 2 Structure Analysis

**Figure 1:**
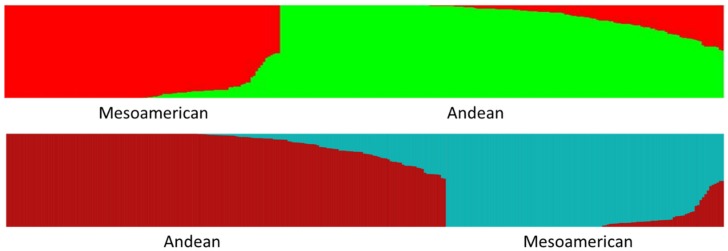
Structure (**top**) and fastStructure (**bottom**) bar charts for *K* = 2 for a snap bean diversity panel. Structure analysis used a 10,000 burn-in and 50,000 Monte Carlo Markov Chain (MCMC) iterations. Andean gene pool is represented by green (Structure) and red (fastStructure) bars, and the Middle American gene pool is shown by the red (Structure) and gray (fastStructure) bars.

There is a surprising degree of agreement between the deep basal branching of phylogenetic trees, Structure analysis, and multiple other clustering methods regarding the splitting of genotypes into either the Andean or Middle American gene pools, although much of the other basal branching can be confused. This natural fault line is evident even for genotypes with a high degree of admixture. A *K* = 2 Structure analysis divided the genotypes into 181 predominately Andean types (>50%) and 114 predominately Middle American types (>50%) (Figure 1 and Figure S1, Table S2). The results for discriminate analysis previously done on a smaller set of genotypes [17] and for K-means clustering (data not shown) are very similar to the Structure result with only four exceptions for genotypes whose level of admixture is very close to 50%. Discriminate analysis identified ‘Coloma’, ‘Esquire’, ‘OR 5402’, and ‘Booster’ as predominately from the Andean gene pool and K-means clustering also identified these four as predominately from the Andean gene pool, except for ‘Coloma’. The *K* = 2 Structure analysis, on the other hand, identified these four as slightly more Middle American. In addition to making apparent the natural division present in common bean, the *K* = 2 Structure analysis also indicated the ratio of mixing between the Andean and Middle American gene pools with 80 of the 246 snap genotypes (32.5%) containing a degree of intermixing greater than 10%, and 52 of the 246 snap genotypes (21.1%) containing a degree of intermixing greater than 20%. Fourteen snap bean genotypes contained nearly equal proportions of both gene pools: ‘91-3982’, ‘Coloma’, ‘Fortex’, ‘Banga’, ‘Booster’, ‘Celtic’, ‘Esquire’, ‘OR 1604M’, ‘OR 5402’, ‘OR 5630’, ‘OR 91G’, ‘Redon’, ‘Selecta’, and ‘Stayton’. From the perspective of an either/or categorization that does not take admixture into account, a *K* = 2 Structure analysis showed that 84 of the 246 (34%) had predominantly Middle American ancestry. When this was broken down by geographic region and plant habit, it became apparent that snap bean gene pools were unequal in their heritage and mixing. This inequality can be seen in the BeanCAP diversity panel that derives primarily from commercial snap beans bred in Europe and North America. Whereas snap beans overall were 34% Middle American in origin based on an either/or categorization in a *K* = 2 Structure analysis, the BeanCAP snap beans (bush habit subset) contained only eight predominantly Middle American origin bean lines out of 141 total bean lines (6%). In addition, approximately 46% of the bush habit BeanCAP were intermixed in their origins at a level higher than 10% according to a Structure analysis compared to only 33% of all snap beans in this study. In stark contrast to the heavy Andean influence found in the BeanCAP, the Chinese genotypes included in this study appeared to be much more skewed towards the Middle American gene pool at 47 out of 55 (87%) Middle American genotypes according to an either/or categorization. The Chinese genotypes also appeared to be much less intermixed than the BeanCAP based on the number of genotypes with greater than 10% intermixing according to a Structure analysis: 5 out of 55 (9%). The Spanish snap beans had both similarities and differences with the BeanCAP and Chinese snap beans. Spanish snap beans, like the BeanCAP, are heavily skewed towards the Andean gene pool with 16 of 19 genotypes (84%) typed as Andean by an either/or categorization. On the other hand, Spanish snap beans appeared to have a much lower level of intermixing based on a Structure analysis with only 3 of 19 (16%) containing greater than 10% intermixing. Further parallels of an intermediate nature can be discerned between Chinese genotypes and pole beans of North American and European descent (excluding Spanish materials). Similar to the Chinese materials, 26 of 31 (84%) pole snap genotypes that excluded the Chinese and Spanish materials was of Middle American origin according to an either/or categorization. Seven of 31 (23%) had a level of mixed heritage greater than 10%. These results are summarized in Table 2.

The dry beans utilized in this study were primarily landraces from the USDA germplasm collection, but three were heirlooms. The dry beans were, by design, divided fairly evenly between Middle American and Andean gene pools: 30 to 19 or a 61% to 39% split using an either/or categorization. None of the dry beans in this study had greater than 20% intermixing according to the Structure analysis, but intriguingly, the majority did show some lesser degree of intermixing. The Structure analysis showed that 4 of 49 dry beans (8%) used in this study had a mixed heritage above 10% but below 20%. The origin of this mixed heritage is unclear. The results from the dry beans are summarized in Table 2.

### 3.2. Expected Heterozygosity and Percent Admixture

Expected HET values varied from 0.20 to 0.36 on a zero to 0.5 scale, with zero indicating that all SNP positions were monomorphic. The high HET value for the dry bean subset showed that this collection was relatively higher in rare alleles and genetic diversity. When all snap beans were combined from multiple geographic locations, the HET value was nearly as high as the dry beans. The lowest HET value were seen in the Spanish collection, although HET values are modulated by population size and the very small *n* of this collection likely reduced the HET value. The results for HET calculations are summarized in Table 2.

### 3.3. Phylogenetic Analysis

**Figure 2:**
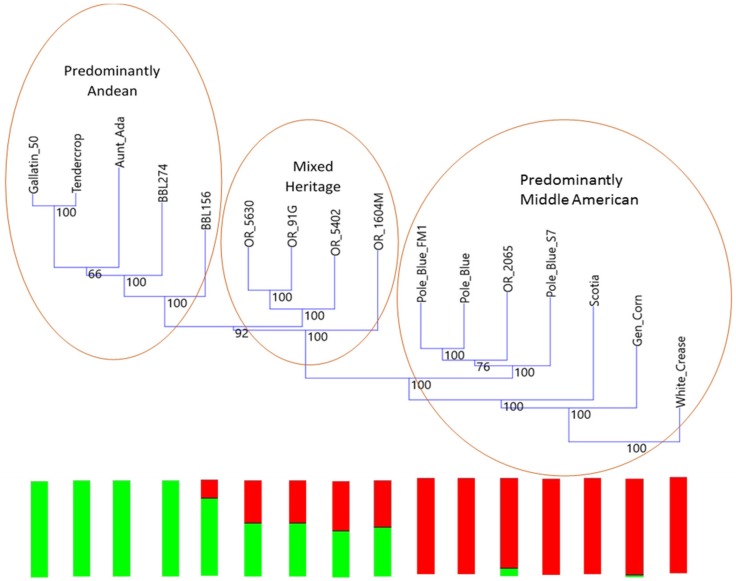
Phylogenetic tree of Blue Lake lines. Bootstrap values are shown at the base of each branching as a percent. ‘Aunt Ada’, ‘Tendercrop’, and ‘Gallatin-50’ were added as Andean outgroups. ‘Scotia’, ‘Genuine Cornfield’, and ‘White Creaseback’ were added as possible progenitors to the Blue Lake lines. All other lines are identified as Blue Lake. The tree was rooted in ‘White Creaseback’. The box plots below the phylogram show the *K* = 2 Structure analysis of mixing between Middle American heritage, shown as red, and Andean heritage, shown as green.

A phylogenetic tree was generated showing Blue Lake materials in relationship to three putative progenitors of the Blue Lake lines and three Andean lines as an outgroup (Figure 2). Baggett and Lucas had proposed ‘Scotia’, ‘Genuine Cornfield’, or ‘White Creaseback’ as possible progenitors to Blue Lake [23]. Based on the close proximity of ‘White Creaseback’ to ‘Pole Blue Lake’ on a biplot of the first and second axes of the PCoA, ‘White Creaseback’ was used to root the phylogenetic tree. The Andean materials included ‘Aunt Ada’ as a pole snap bean that is closely allied to Andean dry beans and to bean race Chile. ‘Gallatin-50’ and ‘Tendercrop’ were also included as exemplary materials of an extremely close genetic relationship because ‘Gallatin-50’ was a white seeded selection out of ‘Tendercrop’. The Phylogram did not show any clear lineage between the proposed progenitors and the Blue Lake lines, but the results also did not exclude them either. Perhaps more interesting than the progenitors is the clear progression of increasing mixing with Andean materials that is apparent from right to left in the phylogram. On the right of the phylogram are the earliest results of breeding in the 1950’s and 1960’s for improved pole snap beans, such as ‘Pole Blue Lake FM-1’. Also among the pole types is the earliest bush Blue Lake bean, ‘OR 2065’, that was the first selection of a cross between a pole Blue Lake type bean and a bush bean. In the middle of the phylogenetic tree are modern commercial bush Blue Lake materials that are still widely planted in Oregon, such as ‘OR 5630’. Finally, to the left can be seen products of modern breeding, such as ‘BBL156’ and ‘BBL274’ whose traits are not congruent with other Blue Lake materials. Included within the figure and below the phylogram are the bar graphs from the *K* = 2 Structure analysis showing the degree of intermixing between the Middle American and Andean centers of domestication. These bar graphs show that the earliest products of Blue Lake breeding were almost entirely Middle American in origin but widely grown materials of a more modern derivation contain a significant Andean admixture and other materials of modern derivation with incongruent Blue Lake traits appear to be almost entirely Andean in origin.

**Figure 3:**
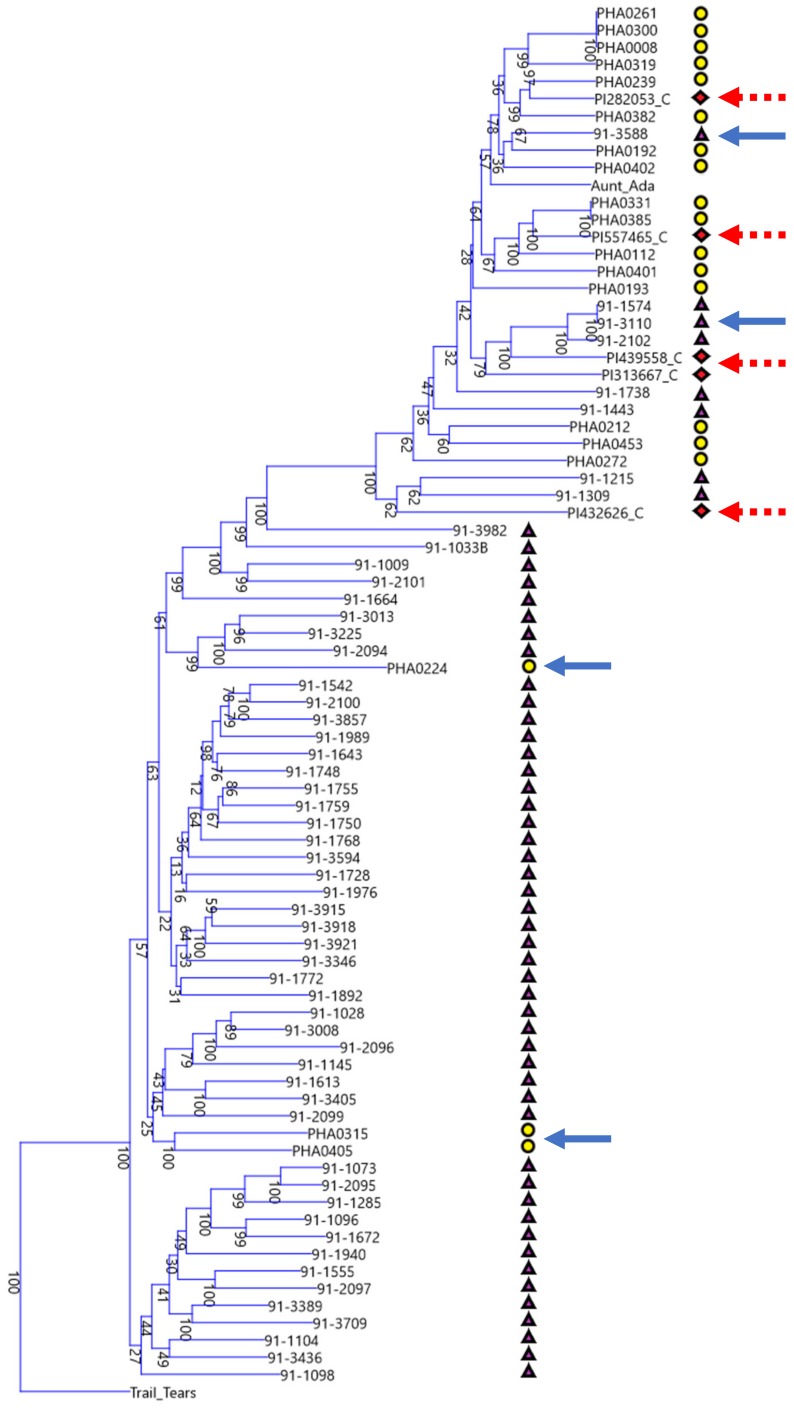
Phylogenetic tree of Spanish, Chinese, and race Chile lines. Also included are the American heirloom types, ‘Cherokee Trail of Tears’ and ‘Aunt Ada’. Bootstrap values for 1000 samplings are shown at the base of each branching as a percent. The tree is rooted in the ‘Cherokee Trail of Tears’ genotype. Phylogenetic connections between Chinese and Spanish bean lines are indicated with a blue arrow. Phylogenetic connections between race Chile dry beans and Chinese and Spanish snap beans are indicated with a dashed red arrow. Yellow circles indicate Spanish origin lines. Purple triangles indicate Chinese origin lines. Red diamonds indicate race Chile lines.

The materials contained in the Misión Biológica de Galicia collection are believed to be very close to the original materials brought from the Americas during the Columbia Exchange and the Chinese materials collected by Michael Dickson may also be of a very old derivation. The connection between these two germplasm collections seems pertinent to understanding the origins of snap traits in world collections of germplasm and to understanding how beans spread and how they were selected. A phylogram containing both geographic collections was generated (Figure 3). The dry beans that were identified in the literature as being of race Chile were also included because this bean race was the most informative in forming clear lineages with snap bean genotypes. The results of this phylogram show clear connections between Spanish and Chinese bean lines as well as to race Chile. In two instances within the Andean gene pool branching, the race Chile landraces fall within the same clade as Chinese materials at a high bootstrap value, and in two further instances the Spanish materials fall within the same clade as race Chile landraces. There are also two instances in the Andean gene pool branching in which Spanish and Chinese lines either fall within the same clade or, as is the case with ‘91-1574’, ‘91-3110’, ‘91-2102’, ‘91-1738’, and ‘91-1443’, appear to be basal to 14 Spanish lines. In two instances in the Middle American gene pool branching, Chinese and Spanish materials can be found in the same clade.

### 3.4. Principal Coordinates Analysis

**Figure 4:**
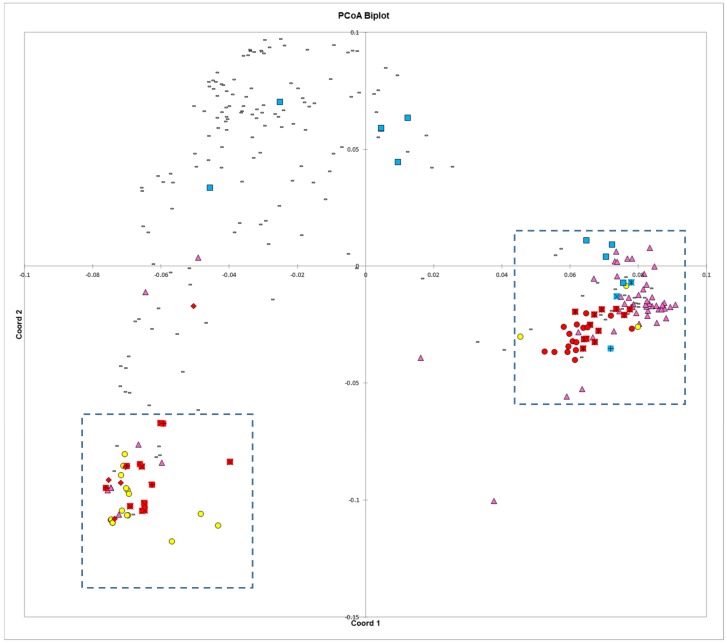
Biplot of the first two axes of a principle coordinates analysis of 295 dry and snap bean accessions. Symbols are as follows: Yellow circles—Spanish lines; purple triangles—Chinese lines; red diamonds—race Chile dry beans; red plus sign—race Nueva Granada; red ‘X’ signs—race Peru; red circles—race Mesoamerica/race Guatemala; red asterisks—race Durango/race Jalisco; blue squares—Blue Lake lines; blue asterisk—‘White Creaseback’; blue ‘X’ sign indicates ‘Genuine Cornfield’; and blue plus sign indicates ‘Scotia’.

**Figure 5:**
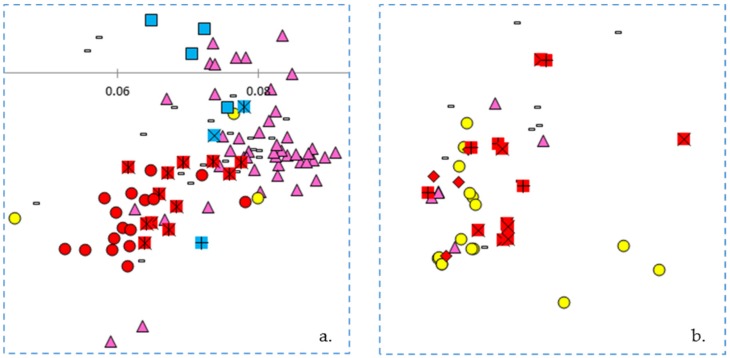
Enlargements of the regions in boxes in Figure 4. (**a**) Middle American accessions, and (**b**) Andean accessions. Symbols are described in Figure 4.

The first principal coordinate axes divided the Middle American and Andean gene pools and represented more than a third of the variation (36.3%). The second principal coordinate axes represented nearly half the total variation in combination with the first axes (45.7%). All other axes individually accounted for 4% of the variation or less. The Chinese lines heavily clustered on the right side of the biplot where genotypes of the Middle American gene pool predominate and the Spanish lines clustered primarily on the left side of the biplot where the Andean gene pool predominates (Figure 4). Blue Lake bean varieties were found in an arc from right to left showing the levels of intermixing with Andean types that has taken place. Of the three proposed progenitors to the Blue Lake lineage, ‘White Creaseback’ showed the closest proximity and therefore the greatest similarity to ‘Pole Blue Lake’. Most snap beans were located in an intermediate space found primarily in the upper left quadrant (Cartesian quadrant II). Two tight clusters found at either end of the biplot represented the poles of the Andean and Middle American gene pools. This is also where the dry bean landraces are located on the biplot. An enlargement of the tight Middle American gene pool cluster found primarily in Cartesian quadrant IV shows close proximity between race Mesoamerica and race Durango/Jalisco to several Chinese genotypes (Figure 5a). In one instance, a Chinese and Spanish genotype nearly overlap each other (Figure 5a). There is also a close proximity between ‘Pole Blue Lake’, ‘White Creaseback’, and Spanish line ‘PHA0315’. On the other side of the biplot is a tight cluster of Andean gene pool genotypes that are enlarged in Figure 5b. In two instances there is close proximity between a member of race Chile and Spanish lines or Chinese lines, but there is also a close proximity between members of race Nueva Granada to a Spanish line, a Chinese line, and to heirloom bean ‘Meraviglia’.

### 3.5. Structure and fastStructure Analysis, K = 8 and 9

fastStructure arrived at an optimum *K* of 8 based comparison of marginal likelihood values. Wilcoxon nonparametric tests for the Structure analysis with 10,000 burn in and 50,000 MCMC iterations revealed significant differences in likelihood value between *K* = 8 and 9 (Prob. ≥ 0.0029, two-sided exact test), but not for *K* = 9 and 10 (Prob. ≥ 0.779).

**Figure 6:**
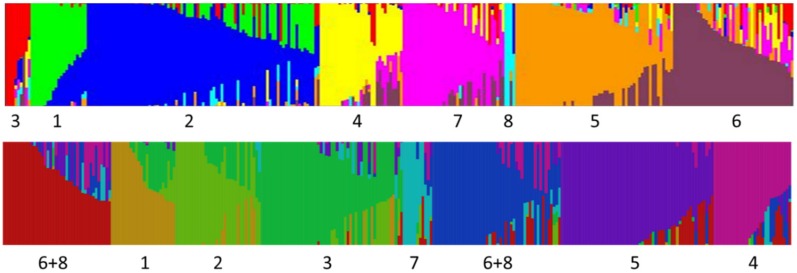
Structure (**top**) and fastStructure (**bottom**) analysis of a snap bean diversity panel for *K* = 8. Structure analysis conducted with 500,000 burn-in and 750,000 MCMC iterations. Numbers below graph correspond to group numbers in Table 3. Color of bars are arbitrarily assigned to each of eight groups (For Structure bar chart, group 1 = green, 2 = blue, 3 = red, 4 = yellow, 5 = orange, 6 = dark red, 7 = purple and 8 = light blue). Solid bars indicate an individual with no admixture while bars with mixed colors indicate admixture with one or more other groups represented by the appropriate color. Height of the bar is proportional to the amount of admixture.

**Figure 7:**
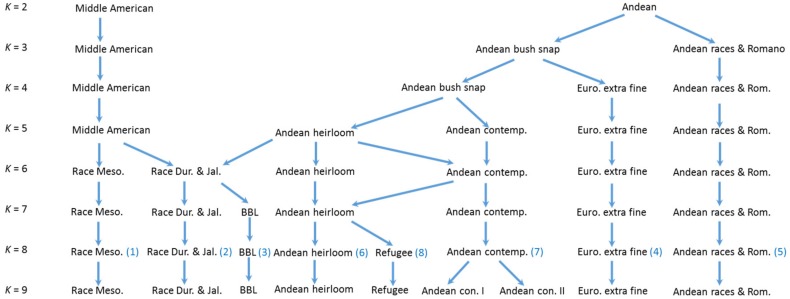
Gene pool divisions from a Structure analysis from *K* = 2 to 9 of a diverse set of snap bean accessions. Diagram shows the *K* level when specific gene pools were observed. Numbers in () at *K* = 8 correspond to the eight groups identified in Table 3. For gene pools BBL (group 3) and Refugee (group 8), diagonal arrows indicate the shift in gene pool identity as *K* increases.

Snap and dry bean accessions could be classified into eight groups based on the 500,000 burn-in– 750,000 iteration Structure analysis for *K* = 8 (Table 3 and Table S3, Figure 6 and Figure S2). These consisted of (1) Race Mesoamerican and most race Guatemala dry bean accessions and a few pole bean accessions, (2) Most race Durango and race Jalisco dry beans along with the majority of remaining Middle American pole beans as well as most Chinese accessions, (3) Bush blue lake green beans, (4) European small-seeded (whole bean or extra fine) snap beans, (5) Andean races (Chile, Peru and Nueva Granada) and large, flat-podded (Romano) pole and bush beans, including most Spanish accessions, (6) Andean origin historical American bush snap beans and their derivatives, (7) Andean origin contemporary American bush snap beans, and (8) a small group of Refugee type half-runner and pole beans. We also examined the gene pool grouping from *K* = 2 to 9 (Figure 7) to examine the stability of groups. All groups except 3 and 8 showed a uniform lineage. In the case of group 3 (Bush blue lake), they were initially included in the Andean gene pool, but at *K* = 6 were included in group 2 before becoming their own distinct lineage at *K* = 7. Group 8 (Refugee types) shifted between Andean groups before showing a distinct grouping at *K* = 8. At *K* = 9, groupings were nearly identical except that a small group of accessions was partitioned from group 7. These accessions showed substantial admixture especially with group 4 but in general show less than 60% identity within this group. While statistically, *K* = 9 appears optimal, the difference from *K* = 8 is trivial and biologically *K* = 8 is more sensible and as such, results from the best *K* = 8 run with 500,000 burn in and 750,000 iterations is used for the remainder of the results and discussion (Figure 6 and Figure S2, Table S3).

Group 1 included all but one each the race Mesoamerica, Durango and Guatemala dry bean accessions, one additional dry bean (Guatemala) and five heirloom pole snap beans. Both ‘Scotia’ and ‘Cherokee Trail of Tears’ were part of this group but showed significant admixture (0.323 and 0.472, respectively) with group 2. In addition to most race Durango all Jalisco dry bean accessions, 47 Chinese, one Spanish accession and one additional dry bean were part of group 2. Among pole snap beans, 23 belonged to group 2 including all three pole Blue Lake accessions, ‘Kentucky Wonder’, ‘Genuine Cornfield’ and ‘White Creaseback’. Two Dutch cultivars (‘Dutch Double White’ and ‘Widusa’) and one French cultivar (‘Fortex’) were also included in this group. Many accessions in this group showed admixture with group 1, but with few exceptions, little mixing with Andean accessions. Most significant were ‘Dutch Double White’ with 0.306 admixture with group 4 (European extra fine types), ‘Purple Stripe Menonite’ with 0.442 admixture with group 8 (Refugee types), and ‘Fortex’ showing introgressions from Bush Blue Lake (BBL) (0.212), Refugee (0.065), Andean landrace (0.055) and historical Andean snap beans (0.259). Group 4 consisted of 31 small seeded and podded bush snap beans, most of which have been bred in Europe although a few cultivars are of American origin. This group showed the greatest degree of admixture with BBL, both historical and contemporary Andean bush beans, and some introgression from Andean landraces and Refugee materials. Group 5 included six Chinese and 16 Spanish accessions. All Romano types belonged to this group as did six heirloom snap beans and one Native American dry bean. Admixtures within this group were primarily with historical Andean snap beans. Group 6 consisted of older Andean snap beans and their more contemporary derivatives. Two Chinese accessions belonged to this group but none of the Spanish accessions shared membership. Older accessions included ‘Black Valentine’ (released in 1897), ‘Brittle Wax’ (1900), ‘Contender’ (1950), and ‘Top Crop’ (1950) [32,33]. Admixture was predominantly with Andean landraces, contemporary Andean bush snap beans, European extra fine and BBL types. Group 7 contained newer Andean bush snap beans, with any admixture being with historical Andean snap beans, European extra fine and BBL types. Group 8 consisted of three Refugee accessions and the heirloom ‘Oregon Giant’. The oldest in this group is ‘Corbett’s Refugee’, which was a bean common mosaic virus (BCMV) resistant selection out of ‘Stringless Green Refugee’, which traces back to the mid-19th century. ‘Oregon Giant’ appears to have been derived from a cross between a Refugee type and a Middle American pole bean with approximately 50% contribution from each.

## 4. Discussion

### 4.1. K = 2 Structure Analysis

The *K* = 2 Structure analysis provides a perspective that is strongly focused on the two overarching Andean and Middle American gene pools present in common bean. This perspective can shed light upon the differences and similarities between the geographic arenas of China, The Iberian Peninsula, Europe, and North America. The *K* = 2 Structure analysis shows that the commercial beans in the BeanCAP diversity panel are both skewed towards the Andean center and heavily intermixed with nearly half containing an admixture to their predominate type of greater than 10% and about a third containing an admixture greater than 20% (Table 2). This likely reflects the work of plant breeders to intercross lines for selection of new trait combinations, but a small portion of this intermixing could also reflect accidental outcrosses in an on farm context, although outcrossing rates in common bean are known to be extremely low. In comparison to the BeanCAP, the rates of intermixing were significantly lower for heirloom pole beans of Europe and North America and for Spanish bean lines, and were lower still for Chinese bean lines and dry beans. This indicates that these bean lines have undergone much less intercrossing by breeders. The Chinese and American heirlooms beans also contained much higher percentages of predominately Middle American germplasm, whereas commercial materials from Europe and North America as well as the Spanish collection are predominately Andean. The high degree of Middle American genetic background in American snap heirlooms may reflect the proximity of Central America to places such as the Southeastern United States.

### 4.2. Expected Heterozygosity

Expected heterozygosity was also analyzed to gain a sense of the genetic diversity present in these germplasm collections. This analysis showed that rare alleles were present at higher rates in the dry beans than in any other collection. This result is consistent with the notion that snap beans were derived from dry beans and that snap beans may have undergone a genetic bottleneck. But it also showed that the cumulative diversity of all the snap beans, including Chinese, Spanish, American/European pole beans, and the BeanCAP were nearly as diverse as our selected dry bean subset containing both centers of domestication and all bean races. This shows that a broader collection of snap beans or a carefully chosen core collection at a gene bank could capture a significant amount of genetic diversity in snap beans. There is a difference in the size of *n* between the dry beans and all snap beans combined, and this difference in *n* would tend to reduce the HET value for dry beans, but this is counterbalanced by the fact that the dry beans were handpicked to be as diverse as possible with all bean races represented. An interesting side-light to this analysis is the surprising diversity of European and American heirloom pole beans (all other pole snap) which was higher than any other subset of snap beans.

### 4.3. Phylogenetic Analysis

The Blue Lake phylogenetic tree shows the degree to which the original Blue Lake genetic background has been altered by breeding efforts over the years. This represents two opportunities for future breeding in the Blue Lake background. First, it suggests a relatively straightforward method for monitoring changes to the genome by gauging the level of admixture. Second, it suggests one path forward for improving Blue Lake, namely returning to the original pole habit materials for the original quality traits that first made Blue Lake beans popular.

The phylogenetic tree of Chinese, Spanish, and race Chile bean lines shows that these disparate geographic centers of secondary diversity (i.e., the Iberian Peninsula and China) are actually connected genetically as can be seen in the multiple shared clades in both the Middle American and Andean gene pools. Despite the geographic distance, this should not be entirely surprising. There are plausible trade routes out of the Americas during the early years of the Columbian Exchange, such as trade through the Spanish colony of the Philippines and then through the Portuguese colony of Macau in modern day China, and day length adaptations that overlap in parts of China and the Iberian Peninsula. According to Singh and colleagues [5], bean race Chile is centered on modern day Chile at a latitude of approximately 30° to 40° in its central part, which overlaps favorably with the roughly 36° to 43° latitude of the Iberian Peninsula and with the Northern parts of China. Yet in spite of these overlaps and cladistic connections, there are also stark differences. When viewed through the lens of either/or groupings, the Chinese materials are overwhelmingly derived from the Middle American gene pool but the Spanish materials are overwhelmingly derived from the Andean gene pool. This can be seen in the disproportionate split between Chinese and Spanish accessions shown in the phylogram centered at the branch point just below ‘91-3982’. Just as shared trade routes and shared day length adaptations at similar latitudes may explain the similarities, differing trade routes and the differing day length adaptations of Southern China in comparison to Chile and the Iberian Peninsula may explain the strong influence of the Mesoamerican gene pool on the Chinese lines.

### 4.4. Principal Coordinates Analysis

The principal coordinates analysis supports many of the findings of the phylogenetic trees. The Blue Lake lines are spread out across the PCoA biplot in a manner that mirrors the phylogenetic tree. The Blue Lake varieties that contain the most Andean admixture are furthest to the left on the phylogram where the Andean genotypes reside and also are the furthest to the left on the PCoA biplot where the Andean gene pool genotypes cluster (Figure 4). Similarly, the mixed genotypes are found in an intermediate position and the purest genotypes from the earliest days of Blue Lake breeding are closely clustered with the genotypes of the Middle American gene pool. The two enlarged clusters from the biplot also verifies and validates that close connections between Chinese and Spanish lines apparent in the phylogram and the connections to race Chile.

The principal coordinates analysis also goes beyond the phylograms and gives more information on the relationships between these different collections and races. Whereas the phylogram does not resolve the closest relative to ‘Pole Blue Lake’ among ‘Scotia’, ‘Genuine Cornfield’, and ‘White Creaseback’, the PCoA biplot seems to give a clear ranking of similarity with ‘White Creaseback’ as closest to ‘Pole Blue Lake’ and Scotia as the furthest. The PCoA biplot also shows a high degree of similarity between ‘Pole Blue Lake’ and Spanish line ‘PHA0315’. Furthermore, the PCoA biplot not only confirms the cladistic connections identified in Figure 3, but also shows that race Nueva Granada is similar to at least one Chinese and Spanish line respectively as well as to one American heirloom pole bean. Also apparent in the biplot is the similarity of dry bean members of race Mesoamerica/Guatemala and race Jalisco/Durango to some Chinese lines.

### 4.5. Genetic Structure of Common Bean

Our analysis was only partially successful in differentiating races of common bean. Races from different centers of origin were reliably separated, but within centers, only Mesoamerican/Guatemalan races were distinct from Durango/Jalisco in the Middle American gene pool. This may be a function of the markers (SNPs) used in this study as other studies have relied on RAPD and SSR markers as well as phenotypic assessments.

One interesting finding is that five of the snap bean groups designated by Structure appear to be unique and separate from the dry beans included in the study. In some cases, this distinctness may indicate an independent lineage and unique alleles in particular groups (such as Refugee types), but more likely, separate grouping is a function of unique combinations of alleles. We have not been able to resolve in this study whether snap beans represent a novel source of genetic variation for dry beans, but there at least appears to be unique combinations of alleles that could assist dry bean breeders in crossing center of domestication barriers.

Some dry bean races showed small, but significant admixture whereas many snap beans within the same *K* group showed no mixing. Based on ancestry, we would have expected the dry bean races to have distinct allelic assemblages and the snap beans belonging to these assemblages to show some admixture. The algorithm as applied in our study seems to indicate that Structure identifies and designates groups based primarily on unique combinations of alleles with less emphasis on unique alleles within populations. For example, we know the ancestry of the Bush Blue Lake types, yet at *K* = 8 Structure designated this group as a distinct population with some individuals having no admixture. A similar pattern can be seen with the European extra fine types. None of these have lineages that trace back to historical materials, but rather, are the result of considerable mixing between other derived snap bean groups.

One of our major findings is that among contemporary snap beans, admixtures between centers of domestication are quite widespread, especially for bush blue lake, European extra fine, and many American Andean snap bean cultivars. Older historical cultivars and landraces do not show the same degree of mixing. The impetus for crossing among different snap types appears to be varied. For example, Middle American and Andean pole types were crossed to bush cultivars to bring a particular type into a bush background more amenable to mechanical harvest. A number of crosses have been made to introgress disease resistances available in dry bean backgrounds of different centers into snap beans (documented in several Plant Variety Protection certificates). Inter genepool crosses among snap types have also been used to transfer quality traits.

### 4.6. Snap Bean Gene Pools

Based on the results of or genetic diversity analysis, we postulate that there have been at least two and possibly up to five independent derivations of snap beans from dry beans. First, Middle American pole beans appear to have been derived from a race Durango/Jalisco ancestor, resulting in a large number of cultivars that center around production along the eastern seaboard of the US. A second derivation occurred from Andean materials and probably race Chile, producing large flat podded types that were introduced into Europe and spread around the world early after the Colombian exchange. Third, round-podded snap beans derived from race Nueva Granada may have been developed. The Andean snap bean groups in our Structure analysis show a large degree of mixing, so it is difficult to point to particular ancestral lines. The fourth distinctive group contains the Refugee types. These are most likely of Andean origin, and in some Structure runs were grouped with other Andean accessions, but we regard them as a distinct and separate group because they are phenotypically distinct from all other snap beans. In their ancestral form, they are late maturing with light green pods on plants with a half runner (type III) growth habit (bush beans are type I and all other pole beans are type IV). In our Structure analysis, this group did not show any particular relationship to any dry bean races, so it is not possible to postulate an ancestral type. Refugee types appear to have been so named because they were brought to the North American by French Huguenots fleeing persecution in Europe [32]. Further complicating the picture, Refugee types have been widely used in breeding in other snap bean groups. For example, ‘Corbett’s Refugee’ has been the primary source of *I* gene resistance to BCMV for most contemporary snap beans. Fifth, there may have been a separate Middle American pole type derived from race Mesoamerica dry beans as typified by ‘Scotia’ and ‘Cherokee Trail of Tears’. Oral histories have revealed that the ‘Cherokee Trail of Tears’ was carried and preserved by the Cherokee during their forced removal from Georgia in the Eastern US to reservations in Oklahoma [17].

We can think of two genetics and genomics tests that could help determine how many independent derivations of snap beans took place. Using a selected set parents that represent the different groups but show little evidence of mixing, these could be crossed in a diallel and the progeny evaluated for complementation of the various snap bean traits. Preliminary studies have indicated that complementation for pod fiber, pod cross-section and suture strings can be observed in a BBL by Andean snap bean cross [34]. Secondly, as candidate genes for snap bean pod traits are identified, these can be sequenced in accessions from different groups to determine whether the same or different alleles are present in different groups.

### 4.7. Geographic Origins of Snap Beans

While there is evidence for more than one independent derivation of the snap bean type, the exact origins remain unknown. One of the main questions is whether snap beans were derived in the New World, or only after dry beans arrived in the Old World. Primary traits involved in the domestication of common bean included reduction in seed dispersal mechanisms (pod shatter), loss of seed dormancy, increase in seed size and seed color diversity, and changes in plant phenology and growth habit. The main driver for evolution in dry bean would be the primary use and consumption of dry seed, with vegetable use only a minor consideration. Only the first trait through loss of some fiber might have led to a snap bean phenotype, but even for this trait, too much fiber loss would compromise threshing ability and lead to a reduction in harvest efficiency. While most cultures around the world would regard immature bean pods as a food for times of shortage, Europe and especially Southern Europe does seem to have had a culture of using immature parts of traditional field crops as vegetables as evidenced by the pre-Columbian exchange vegetable use of peas, bottle gourd, cole crops and several leafy vegetables. Thus, Europeans may have been predisposed to adopting field crops such as dry bean and developing vegetable forms from these. Available evidence suggests that at least some snap bean derivations happened in Europe, but some derivations may have happened in the Americas and were then brought to Europe. In particular, the small subset of Middle American pole beans that appear most closely related to race Mesoamerica may have been developed in the Southeastern US by the Cherokee or related tribes. Pre-Colombian introduction routes for dry beans into North America probably varied based on bean race. Those of race Durango most likely came overland from Mexico and were carried northwards to what is now North Dakota and eastwards into the central and northeastern US. Race Mesoamerica beans originated in Central American and may have come to the US by dispersal to Cuba and hence into Florida, and up the eastern seaboard as far as present day Ontario. The Guanahatabey of Western Cuba were traditional users of small black beans, which were probably introduced directly from Middle America [35], (J. Kelly, MSU, pers. comm.). These materials are genetically distinct, and at least in the case of ‘Cherokee Trail of Tears’, we have an oral history that indicates stewardship, if not origination of the cultivar. That said, the Colombian exchange began at least two centuries prior to the Trail of Tears events beginning in 1838, so it is possible that these types were introduced to Native Americans by early European settlers.

The large flat podded Andean types are another group that may have had its origins in South America rather than in southern Europe. Evidence for this includes the close affinity of these types to dry bean races (especially race Chile) and the fact that some Spanish and Chinese accessions show close similarity. The relatedness of accessions from these secondary centers would suggest that these were derived from the same original source.

## 5. Conclusions

We hope that this research will inform core collection decisions at gene banks and assist in identifying sources of traits for breeding. It is clear from the data that breeders have heavily relied upon limited genetic resources in Europe and North America, and largely ignored genetic resources elsewhere, such as pole snap beans of North American and Europe, China, and the Iberian Peninsula. Obvious barriers exist to introgressing traits from pole habit beans, including undesirable linkage drag, but it may be worth the effort if genetic gain has plateaued and genuinely novel trait combinations are sought. The history of snap beans to date has been complicated with possibly between two and five derivations from dry beans, and multiple bean races represented. Considering that some degree of incompatibility exists between the two centers of domestication, this mixing could be of use to dry bean breeders as a bridge between the centers of domestication and a source of novel trait combinations that have already been selected for fitness in the field.

## Figures and Tables

**Table 1:** Pod characteristics of dry compared to various snap bean types. (Modified from Myers and Kmiecik, 2017 [13]).

Type	Pod Wall Fiber	Pod Cross-Section Shape	Pod Wall Thickness	Pod Suture Fiber (“Strings”)
Dry	High	Flat, oval	Thin	Present
Romano	Low	Flat	Thin	Generally absent
String	Low	Oval, rhomboid, round	Thick	Present
Snap	Low	Oval, round	Thick	Absent

**Table 2:** Heterozygosity (HET) values, domestication origin and degree of admixture in the heritage of different subsets of dry and snap beans utilized in this study.

Population	*n*	HET Value ^a^	>10% Admixture ^b^	>20% Admixture ^c^	Middle American ^d^	Andean ^e^
			%			
All Beans	295	0.36	28.47	17.63	38.64	61.36
All Snap Beans	246	0.34	32.52	21.14	34.15	65.85
All Dry Beans	49	0.36	8.16	0.00	61.22	38.78
Spanish Snap	19	0.20	15.79	5.26	15.79	84.21
Chinese Snap	55	0.27	9.09	3.64	85.45	14.55
All BeanCAP snap	149	0.26	44.97	30.20	10.74	89.26
Bush BeanCAP snap	141	0.24	46.10	30.50	5.67	94.33
All Other Pole Snap	31	0.30	22.58	12.90	83.87	16.13

^a^ Expected heterozygosity measure of genetic diversity. ^b^ The percent of genotypes containing more than 10% admixture to their predominant Middle American or Andean heritage. ^c^ The percent of genotypes containing more than 20% admixture to their predominant Middle American or Andean heritage. ^d^ The percent of genotypes identified by *K* = 2 Structure analysis to be of predominately (>50%) Middle American heritage. ^e^ The percent of genotypes identified by *K* = 2 Structure analysis to be of predominately (>50%) Andean heritage.

**Table 3:** Categories for snap bean types from a Structure analysis of a diversity panel for *K* = 8.

Group	Description
1	Race Mesoamerican and most race Guatemala dry bean accessions; a limited number of heirloom pole bean accessions originally from the SE US.
2	Most race Durango and race Jalisco dry beans; most Chinese accessions; many pole beans originating in the southern US.
3	Bush Blue Lake (BBL) green beans and accessions with BBL heritage.
4	European small-seeded (whole bean or extra fine) snap beans.
5	Andean dry bean races (Chile, Peru and Nueva Granada); large, flat-podded (Romano) pole and bush beans; a few snap beans.
6	Historical American bush snap beans and their derivatives.
7	Contemporary American bush snap beans.
8	Refugee type half-runner and pole beans.

## Supplementary Materials

The following are available online at http://www.mdpi.com/2073-4425/9/12/587/s1, Table S1. Passport information on 295 genotypes utilized in this study of snap bean genetic diversity. Data are a combination of field notes, Plant Variety Protection applications, catalog descriptions and ASHS Cultivar Descriptions for North America (http://cucurbitbreeding.com/todd-wehner/publications/vegetable-cultivar-descriptions-for-north-america/; verified 10/08/18). Accessions are listed in the order in which they were analyzed in Structure and fastStructure. Bean race identifiers are appended to the names of the landrace dry beans. The appended identifiers are J (Jalisco), D (Durango), M (Mesoamerica), G (Guatemala), P (Peru), C (Chile), and NG (Nueva Granada), Table S2. Summary of *K* = 2 Structure analysis and K-means clustering analysis for a snap bean genetic diversity study, Table S3. Summary of *K* = 8 Structure and fastStructure analysis for a snap bean genetic diversity study, Figure S1. *K* = 2 Structure analysis bar chart for 295 snap and dry bean accessions genotyped with 5396 SNPs. Numbering corresponds to column one of Tables S1 and S2, Figure S2. Structure bar chart for *K* = 8 of an analysis of snap bean diversity. Numbers correspond to accession numbers listed in Tables S1 and S2. The original SNP data used in this study has been deposited in the ScholarsArchive@OSU and can be retrieved using the following link: https://ir.library.oregonstate.edu/concern/datasets/3t945x17v.
