# Supplementary material for: Genetic Diversity within Snap Beans and Their Relation to Dry Beans
Source: Genes (Basel). 2018 Nov 28;9(12):587. doi: 10.3390/genes9120587 (PMC6315957; doi:10.3390/genes9120587)
Supplement: Supplementary file 1 [file genes-09-00587-s001.zip › genes-379875-Supplementary figures.docx]

Supplemental Figures


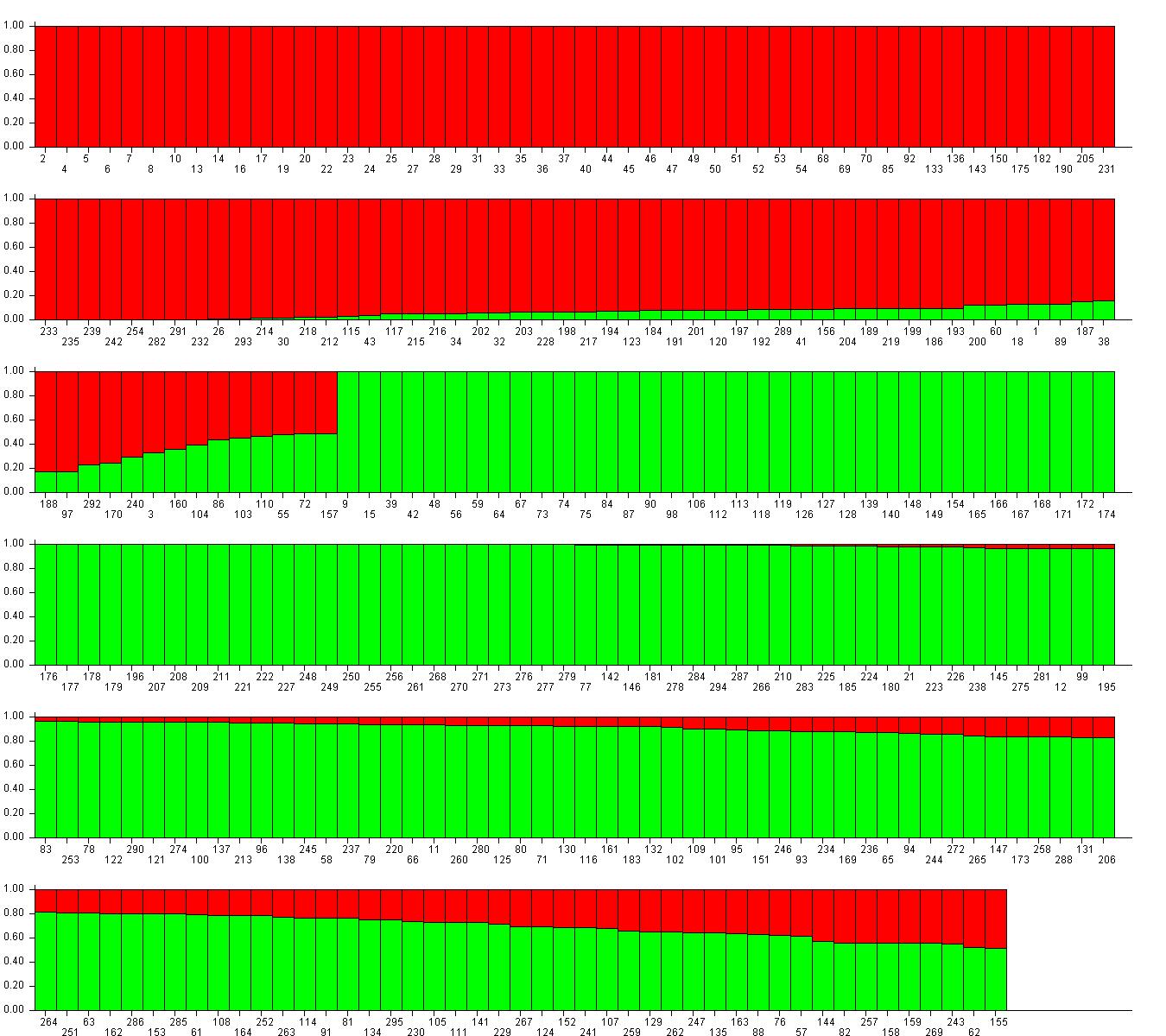


**Figure S1.** K = 2 Structure analysis bar chart for 295 snap and dry bean accessions genotyped with 5396 SNPs. Numbering corresponds to column one of Tables S1 & S2.


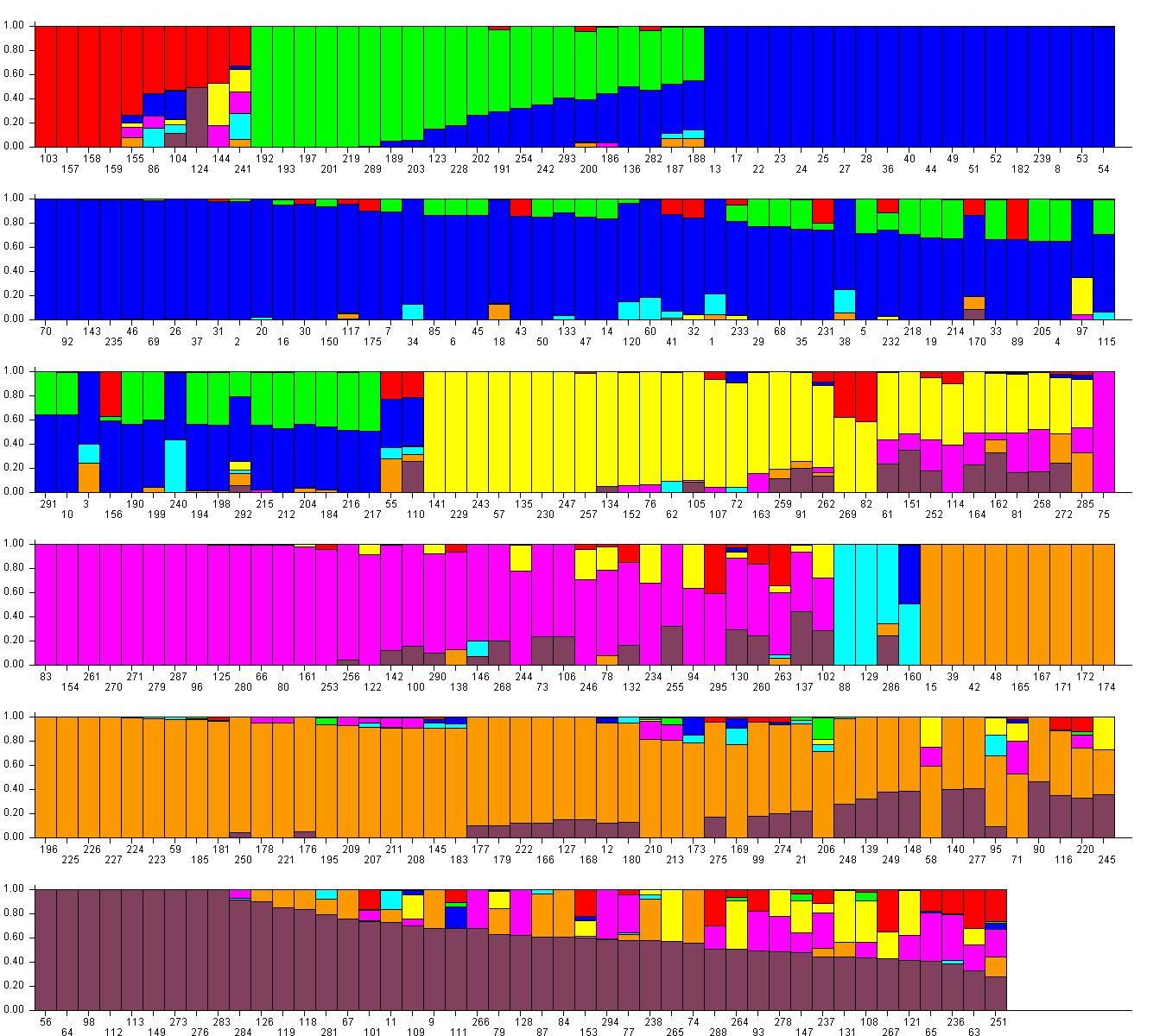


**Figure S2.** K = 8 Structure analysis bar chart for 295 snap and dry bean accessions genotyped with 5396 SNPs. Numbering corresponds to column one of Tables S1 & S2.
